# P130Cas Src-Binding and Substrate Domains Have Distinct Roles in Sustaining Focal Adhesion Disassembly and Promoting Cell Migration

**DOI:** 10.1371/journal.pone.0013412

**Published:** 2010-10-18

**Authors:** Leslie M. Meenderink, Larisa M. Ryzhova, Dominique M. Donato, Daniel F. Gochberg, Irina Kaverina, Steven K. Hanks

**Affiliations:** 1 Department of Cell and Developmental Biology, Vanderbilt University School of Medicine, Nashville, Tennessee, United States of America; 2 Department of Radiology and Radiological Sciences, Vanderbilt University School of Medicine, Nashville, Tennessee, United States of America; 3 Department of Physics and Astronomy, Vanderbilt University, Nashville, Tennessee, United States of America; 4 Vanderbilt University Institute of Imaging Science, Nashville, Tennessee, United States of America; University of Birmingham, United Kingdom

## Abstract

The docking protein p130Cas is a prominent Src substrate found in focal adhesions (FAs) and is implicated in regulating critical aspects of cell motility including FA disassembly and protrusion of the leading edge plasma membrane. To better understand how p130Cas acts to promote these events we examined requirements for established p130Cas signaling motifs including the SH3-binding site of the Src binding domain (SBD) and the tyrosine phosphorylation sites within the substrate domain (SD). Expression of wild type p130Cas in Cas −/− mouse embryo fibroblasts resulted in enhanced cell migration associated with increased leading-edge actin flux, increased rates of FA assembly/disassembly, and uninterrupted FA turnover. Variants lacking either the SD phosphorylation sites or the SBD SH3-binding motif were able to partially restore the migration response, while only a variant lacking both signaling functions was fully defective. Notably, the migration defects associated with p130Cas signaling-deficient variants correlated with longer FA lifetimes resulting from aborted FA disassembly attempts. However the SD mutational variant was fully defective in increasing actin assembly at the protruding leading edge and FA assembly/disassembly rates, indicating that SD phosphorylation is the sole p130Cas signaling function in regulating these processes. Our results provide the first quantitative evidence supporting roles for p130Cas SD tyrosine phosphorylation in promoting both leading edge actin flux and FA turnover during cell migration, while further revealing that the p130Cas SBD has a function in cell migration and sustained FA disassembly that is distinct from its known role of promoting SD tyrosine phosphorylation.

## Introduction

Cell migration is a highly dynamic process during which numerous signals must be integrated to generate a coordinated cellular response. The cell migration cycle consists of several steps including protrusion of the leading edge, adhesion to the substratum, and release of the cell rear (reviewed in [1], [2], [3]). Cell adhesion to the extracellular matrix (ECM) is mediated by integrins, transmembrane receptors that link the ECM to the actin cytoskeleton. These integrin contact sites, commonly called focal adhesions (FAs), not only mediate adhesion, but also form a large protein signaling network that regulates cell migration [4]. Tyrosine kinase signaling is a central aspect of this “integrin adhesome” [5].

**Figure 1:**
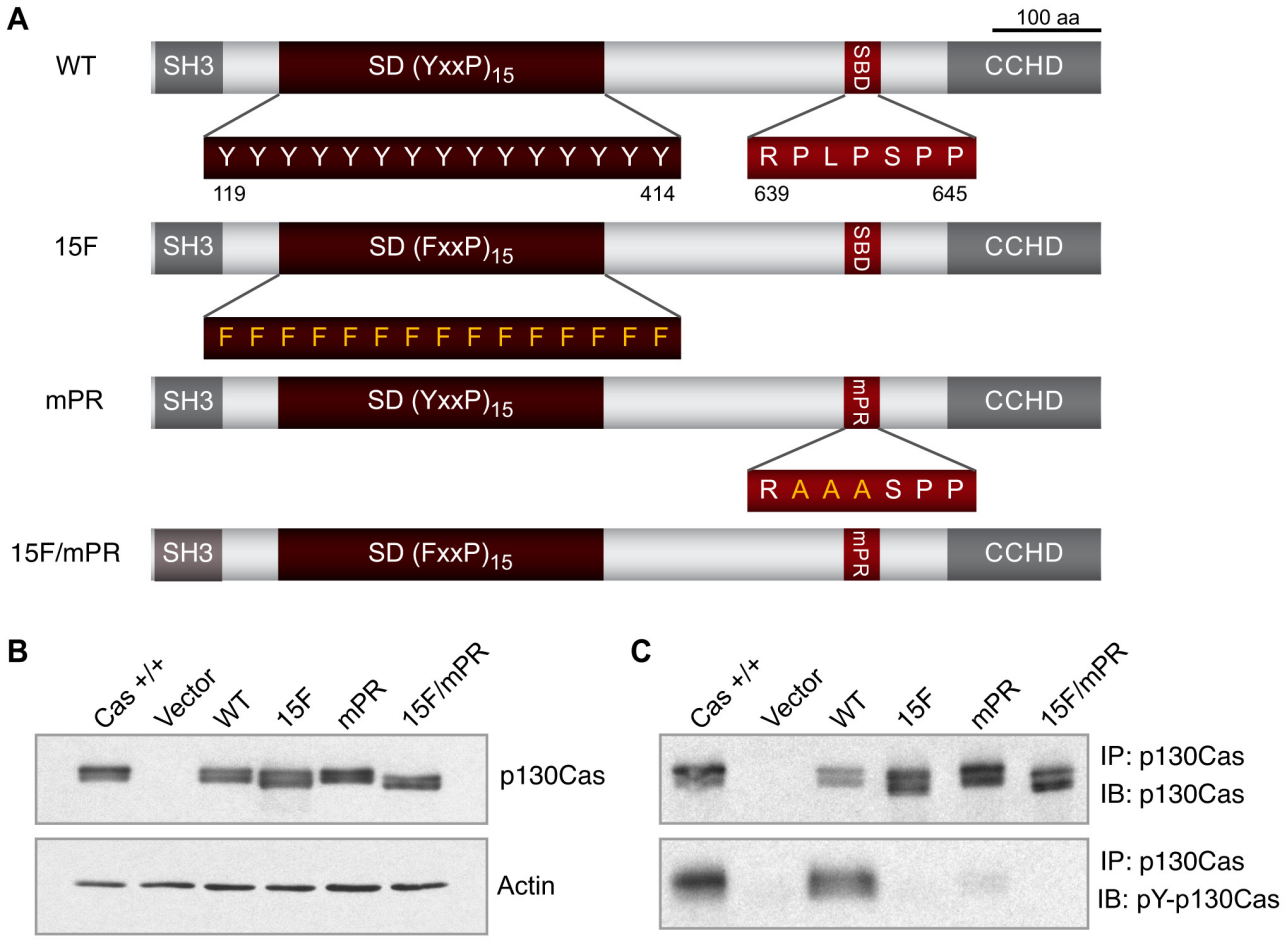
Expression of p130Cas signaling variants in Cas −/− MEFs. (A) Depictions of p130Cas variants used in this study indicating the SH3 domain, substrate domain (SD), Src-binding domain (SBD), and C-terminal Cas-family Homology (CCH) Domain. Shown in addition to p130Cas WT are three full-length signaling variants with amino acid substitutions indicated; 15F, all fifteen YxxP tyrosines in the SD changed to phenylalanine; mPR, proline-rich motif RPLPSPP in the SBD mutated to RAAASPP; and 15F/mPR double mutant. (B) Immunoblot analysis of whole cell lysates of Cas−/− MEFs reconstituted with p130Cas variants. Cas +/+ MEFs were included as a control. Equal amounts of total protein were loaded, 15 µg per lane. WT, 15F, mPR, and 15F/mPR variants are expressed at levels similar to endogenous p130Cas in Cas +/+ MEFs. Actin immunoblot is included as a loading control. (C) Analysis of p130Cas SD tyrosine phosphorylation. Immunoprecipitates (IP) of p130Cas variants were prepared and assessed by immunoblotting (IB). Cas +/+ MEFs were included as a control. Near equal amounts of p130Cas total protein were loaded for each sample and assessed using the same antibody used for immunoprecipitation, top panel. SD tyrosine phosphorlation (SD-pY) was assessed from the same immunoprecipitates using a mixture of phosphospecific antibodies that recognize SD YxxP sites, lower panel.

P130Cas (Crk-associated substrate) is a prominent substrate of the Src tyrosine kinase in the integrin adhesome that has been implicated in the control of cell migration [6], [7], [8], [9]. P130Cas is ubiquitously expressed [10], and the mouse knockout is embryonic lethal with Cas −/− mouse embryonic fibroblasts (MEFs) having disorganized actin stress fibers and defects in cell migration [11]. P130Cas contains multiple conserved protein-protein interaction domains including an N-terminal SH3 domain that interacts with focal adhesion kinase (FAK), a central “substrate domain” (SD), a “Src-binding domain” (SBD) near the C-terminus, and a conserved “C-terminal Cas-family homology” (CCH) domain [7], [8], [9] (Fig. 1). While the SH3 and CCH domains mediate localization to FAs [12], the SD [13], [14], [15] and SBD [16] have direct functions in initiating signaling events.

The SD signals by undergoing tyrosine phosphorylation in order to recruit downstream effectors. The SD is characterized by fifteen YxxP motifs, ten of which can be efficiently phosphorylated by Src [15]. FAK shows no tyrosine kinase activity toward p130Cas, but interacts with the Src SH2 domain to recruit and activate Src to phosphorylate the p130Cas SD [17]. Direct binding of the Src SH3 domain to a proline-rich binding motif in the SBD [16], [17] also recruits and activates Src to phosphorylate the p130Cas SD. Mutational alteration of the Src-SH3 binding motif in the SBD effectively blocks the Src/p130Cas interaction [16], [18] and significantly reduces levels of p130Cas SD tyrosine phosphorylation [19], [20]. The ability of Src to phosphorylate the SD is enhanced by physical extension of the SD, implicating p130Cas as a mechanosensor [21]. P130Cas SD phosphorylation has been detected predominantly at FAs and at the cell periphery in nascent integrin adhesion sites [19], [22]. A p130Cas variant with phenylalanine substitutions of all fifteen SD YxxP tyrosines still localizes to FAs [23], indicating that SD tyrosine phosphorylation is not required for FA localization. After activation by tyrosine phosphorylation, the SD is able to recruit downstream effectors, notably the SH2/SH3 adaptors Crk and Nck that can act to promote protrusion of the plasma membrane [6], [24]. While the recruitment and activation of Src to phosphorylate the SD is the best-characterized signaling function of the SBD, Src activated by binding to the SBD has the potential to phosphorylate other proteins [16], which could represent a distinct signaling function of the SBD.

P130Cas has been implicated in regulating two cellular processes essential to motility: protrusion of the leading edge and FA disassembly. Recruitment of Crk and Nck to the tyrosine-phosphorylated p130Cas SD is implicated in promoting protrusion [13], [14], [24], although direct quantitative measurements of leading edge actin dynamics stimulated by SD tyrosine phosphorylation are still lacking. A study evaluating the turnover of adhesion components in randomly migrating cells implicated p130Cas in regulating FA disassembly, with the apparent rate constant for FA disassembly ∼19-fold lower in Cas −/− MEFs as compared to cells expressing wild type (WT) p130Cas [25]. No studies have addressed the mechanism by which p130Cas promotes FA disassembly.

Here, we utilized Cas −/− MEFs expressing p130Cas signaling variants and live cell imaging to carry out a detailed investigation of the roles of the p130Cas signaling domains in mediating cell migration and the key physical steps of leading edge actin dynamics and FA assembly/disassembly. Our results show that p130Cas SD tyrosine phosphorylation sites are required to stimulate leading edge actin assembly and plasma membrane protrusion and to enhance the rates of both FA assembly and disassembly. Furthermore, we show that the SD and SBD make distinct additive contributions to ensure that FA disassembly proceeds without interruption.

## Results

### P130Cas SD and SBD signaling domains have distinct roles in stimulating cell migration

Cas −/− MEFs stably expressing WT p130Cas versus mutational variants (Fig. 1A) were generated in order to evaluate the contribution of p130Cas signaling domains to cell migration. Three p130Cas mutants were expressed: an SD mutant (15F) in which all fifteen YxxP motif tyrosines were changed to phenylalanines, an SBD mutant (mPR) in which prolines in the SH3-binding motif are changed to alanines, and a double mutant (15F/mPR) in which both the SD and SBD are changed. All p130Cas variants were stably expressed in Cas −/− MEFs using a bicistronic retroviral vector that co-expresses GFP. Empty vector cells, which express only GFP, were also prepared as a control. Sorting the cells for low GFP levels yielded populations where the p130Cas variants were expressed to levels similar to endogenous p130Cas in Cas +/+ MEFs (Fig. 1B). Moreover, all expressed variants gave rise to the electrophoretic mobility isoforms characteristic of p130Cas and the expected SD tyrosine phosphorylation pattern (Fig. 1C). p130Cas WT cells have robust SD tyrosine phosphorylation similar to Cas +/+ cells, mPR cells have a reduced but detectable level of SD tyrosine phosphorylation, and no SD tyrosine phosphorylation is detected in Vector, 15F, and 15F/mPR cells as expected due to the absence of p130Cas protein or SD phosphoacceptor tyrosines.

**Figure 2:**
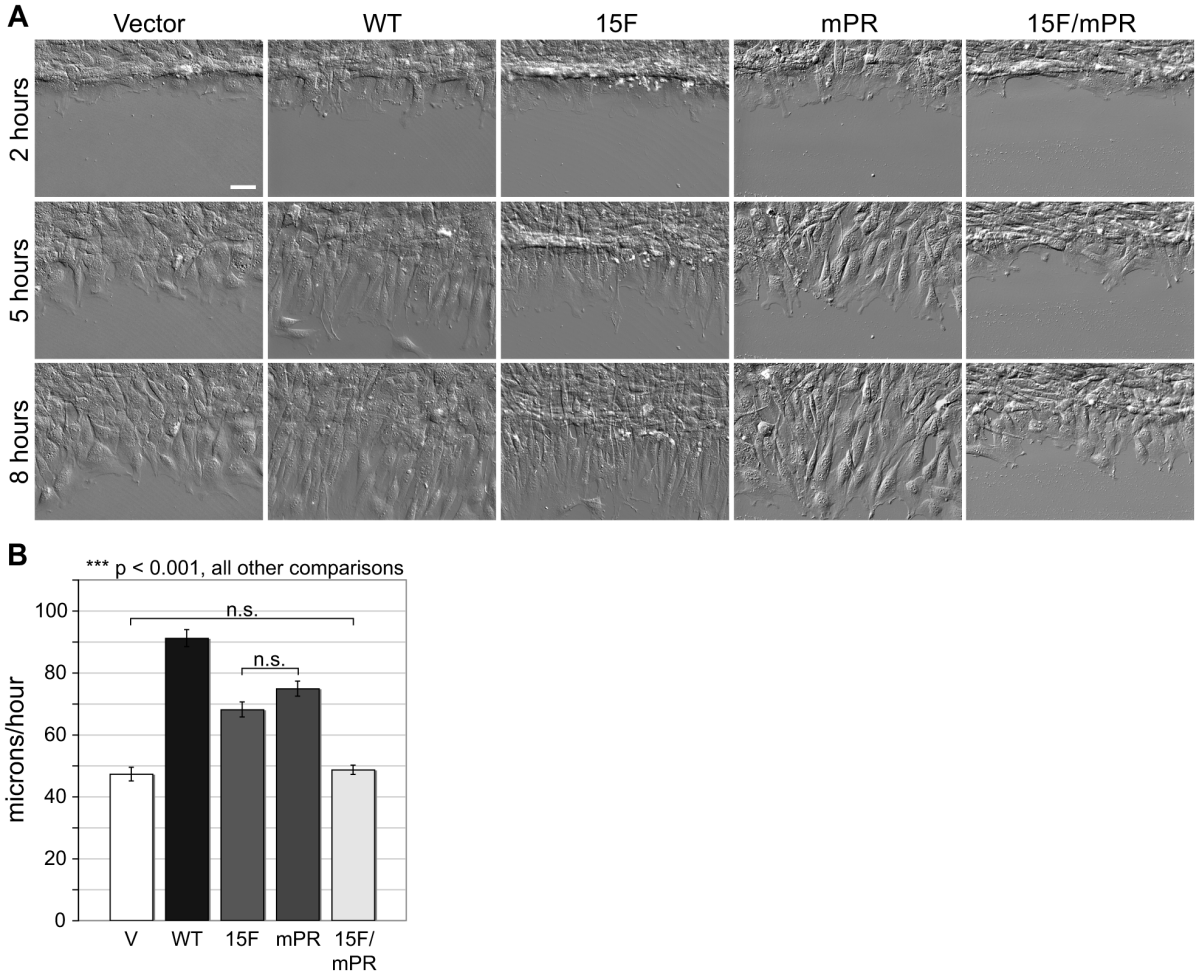
p130Cas SD and SBD signaling stimulate wound healing migration. Monolayer scratch wounds were generated and cells were imaged over 8 hours by live DIC microscopy. (A) Representative images of cells expressing either p130Cas WT, 15F, mPR, or 15F/mPR variants at 2, 5, and 8 hours post wounding. Vector only control cells are also shown. Scale bar is 50 µm. (B) Migration rates were quantified by tracking nuclei of 75 individual cells (25 each from 3 separate wounds) for each cell type. Mean migration rate was calculated as the total distance traveled over the 2 to 8 hour period after wounding. Bars indicate s.e.m. Significance values were determined by one-way ANOVA followed by Tukey-Kramer post hoc testing; n.s. (not significant), ***p<0.001.

These cell populations (WT, 15F, mPR, 15F/mPR, and Vector) were employed to determine the contributions of the two p130Cas signaling domains to cell migration. A monolayer scratch wound assay was used to stimulate migration, and then cells migrating into the wound were imaged over 8 hours using differential interference contrast (DIC) microscopy. Fig. 2A shows representative wound edges at 2, 5, and 8 hours post wounding. Tracking the movement of individual cell nuclei over the 2–8 hour period showed that WT cells migrated into the wound at a mean rate significantly faster (1.9 fold) than Vector control cells (Fig. 2B). The 15F and mPR cells migrated at an intermediate rate, but still significantly faster than Vector cells (1.4 and 1.6 fold, respectively). Only the 15F/mPR cells failed to migrate into the wound faster than the Vector cells. This additive defect indicates that the SBD plays a role in driving cell migration that is distinct from its ability to promote SD tyrosine phosphorylation. We also evaluated individual cells in a replating assay in order to determine if p130Cas SD and SBD signaling facilitated cell spreading. These studies (supplemental Fig. S1) indicated that both p130Cas SD and SBD signaling facilitate the initiation of cell spreading, but do not have obvious effects on the final spread cell area. Thus in addition to promoting cell migration, both p130Cas SD and SBD signaling are implicated in regulating cell adhesion. To gain a better understanding of how the p130Cas signaling domains promote cell migration and adhesion we analyzed the component steps of actin-driven leading edge protrusion and FA dynamics.

### P130Cas SBD-mediated SD signaling is implicated in promoting actin flux at the leading edge lamellipodium

**Figure 3:**
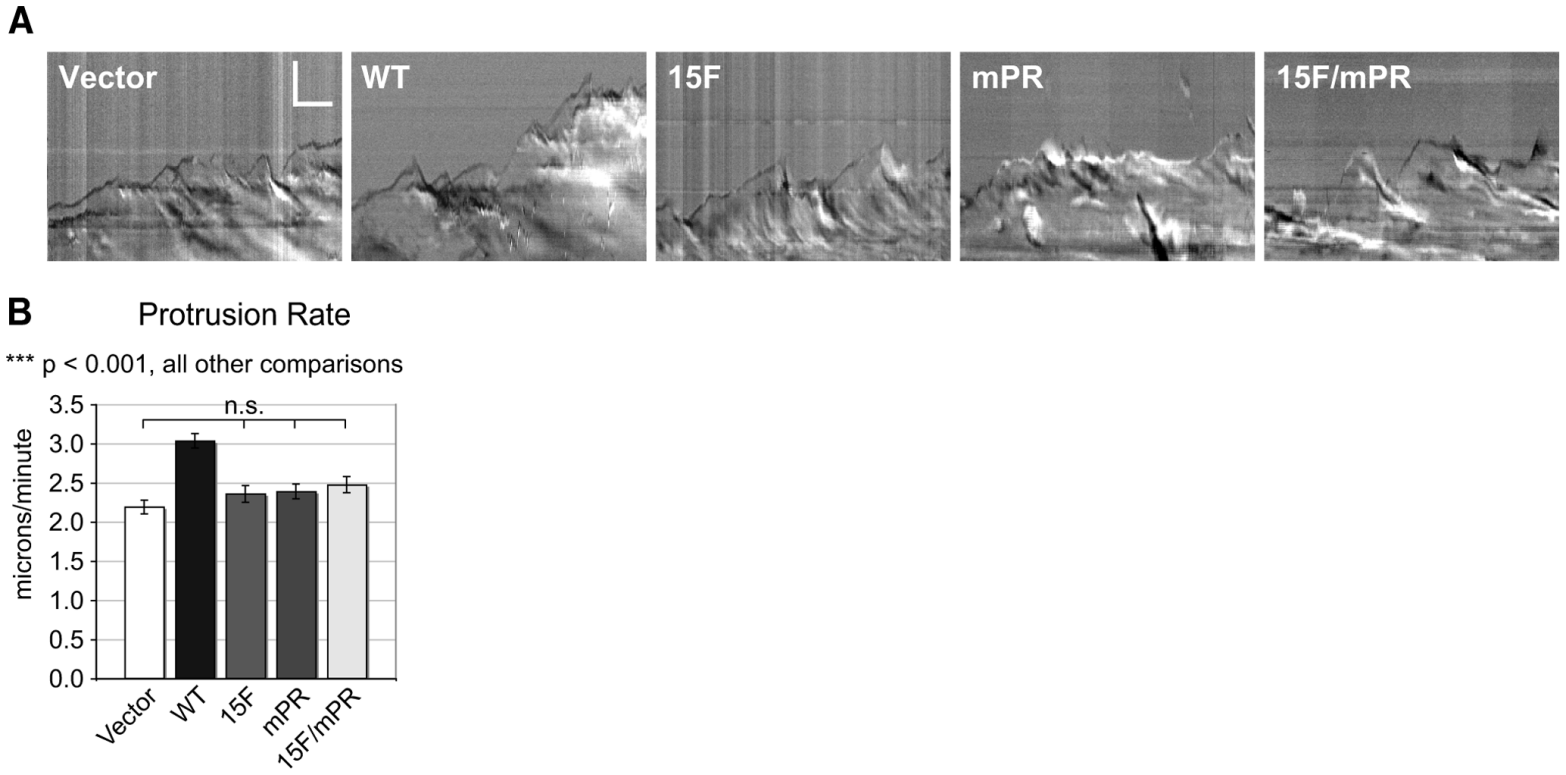
p130Cas SD signaling stimulates leading edge protrusion. Monolayer scratch wounds were generated and DIC images of cells migrating at the wound edge were captured every 3 seconds for 15 minutes and used to evaluate leading edge dynamics using kymography. (A) Sample kymographs representative of mean protrusion values. Vertical scale bar is 5 µm, horizontal scale bar is 2 minutes. (B) Rates of protrusion representing the mean of 60–90 individual cells for each cell type. Leading edge protrusion velocity was quantified by measuring the distance and duration of individual protrusions from 3 kymographs per cell. Bars indicate s.e.m. Significance values were determined by one-way ANOVA followed by Tukey-Kramer post hoc testing; n.s. (not significant), ***p<0.001.

We evaluated how p130Cas signaling impacts plasma membrane protrusion and actin flux at the leading edge of cells migrating at a wound edge using kymography of high temporal resolution DIC images. Fig. 3A shows representative kymographs. Quantitative analysis indicated that cells expressing WT p130Cas have increased protrusion velocities relative to Vector cells and to all three p130Cas signaling variants (Fig. 3B). Thus mutation of the SD is sufficient to reduce the protrusion rate to the levels seen in Vector cells. Since mutation of the SBD also results in impaired SD tyrosine phosphorylation (Fig. 1C) [17], [19], the finding that 15F and mPR cells have similar protrusion rates implicates the primary role of the SBD in protrusion as mediating SD tyrosine phosphorylation.

**Figure 4:**
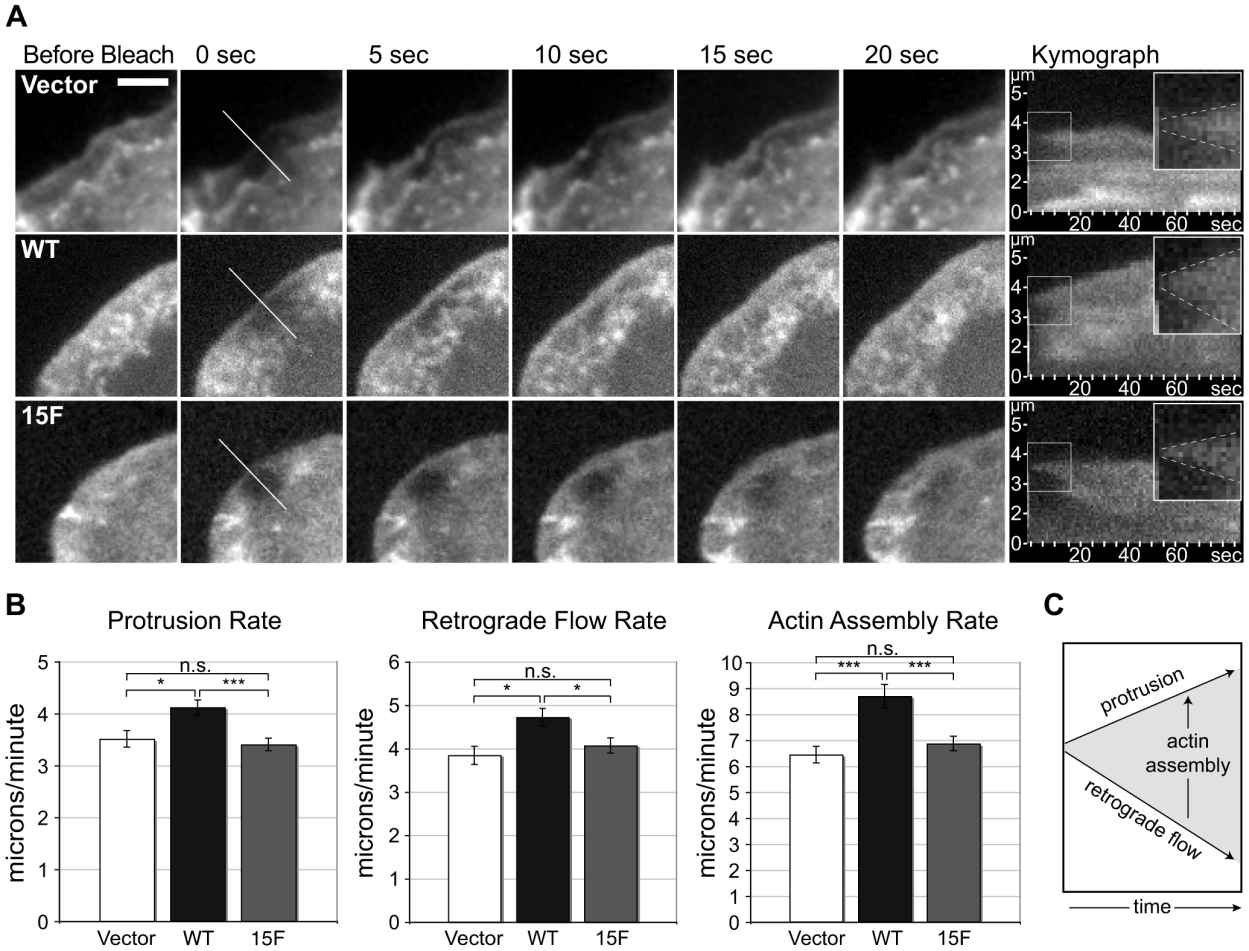
p130Cas SD signaling stimulates actin flux through the leading edge lamellipodium. p130Cas WT, 15F, and Vector only cells expressing a low level of mCherry-actin and migrating at the wound edge were viewed by wide field fluorescence microscopy. The leading edge was photobleached for 15 seconds then images were captured at one-second intervals and analyzed using kymography. (A) Time-lapse images and kymographs (far right) of mCherry-actin representative of mean values for actin assembly. Kymograph inset is a 2× magnification of the boxed area with upper and lower dotted lines marking protrusion and retrograde flow, respectively. Nascent actin assembly is characterized by the area between the dotted lines. Scale bar is 5µm. (B) Rates of protrusion, retrograde flow, and actin assembly representing the mean of 57 to 92 individual cells for each cell type. Bars indicate s.e.m. Significance values were determined by one-way ANOVA followed by Tukey-Kramer post hoc testing; *p<0.05, ***p<0.001. (C) Schematic of protrusion, retrograde flow, and actin assembly rate calculations.

To further study the impact of p130Cas SD signaling on leading edge protrusion, Vector, WT, and 15F cells were transiently transfected to express mCherry-actin and photobleaching of the fluorophore was used to assess actin dynamics. Cells migrating at the wound edge and expressing low levels of mCherry-actin were selected, then a region of the leading edge lamellipodium with active protrusion was bleached to create a reference mark. A kymograph through the bleach region was used to measure actin protrusion velocity and retrograde flow rate, and these values were used to calculate the actin assembly rate (Fig. 4A shows representative examples and Fig. 4C shows a schematic of the rate calculation). Cells expressing WT p130Cas display higher values for all three rates in comparison to Vector cells (Fig. 4B). The cells expressing the p130Cas 15F mutant have protrusion, retrograde flow, and actin assembly rates similar to those in Vector cells. The failure of the 15F mutant to stimulate actin flux implicates SD tyrosine phosphorylation as the primary mechanism by which p130Cas promotes actin assembly at the leading edge. Note that the protrusion rate values shown in Fig. 3 are somewhat lower than those shown in Fig. 4B, which is due to the fact that only a subpopulation of actively protruding cells were selected for bleaching in the latter analysis.

### P130Cas SD and SBD signaling domains have distinct roles in shortening FA lifetimes

**Figure 5:**
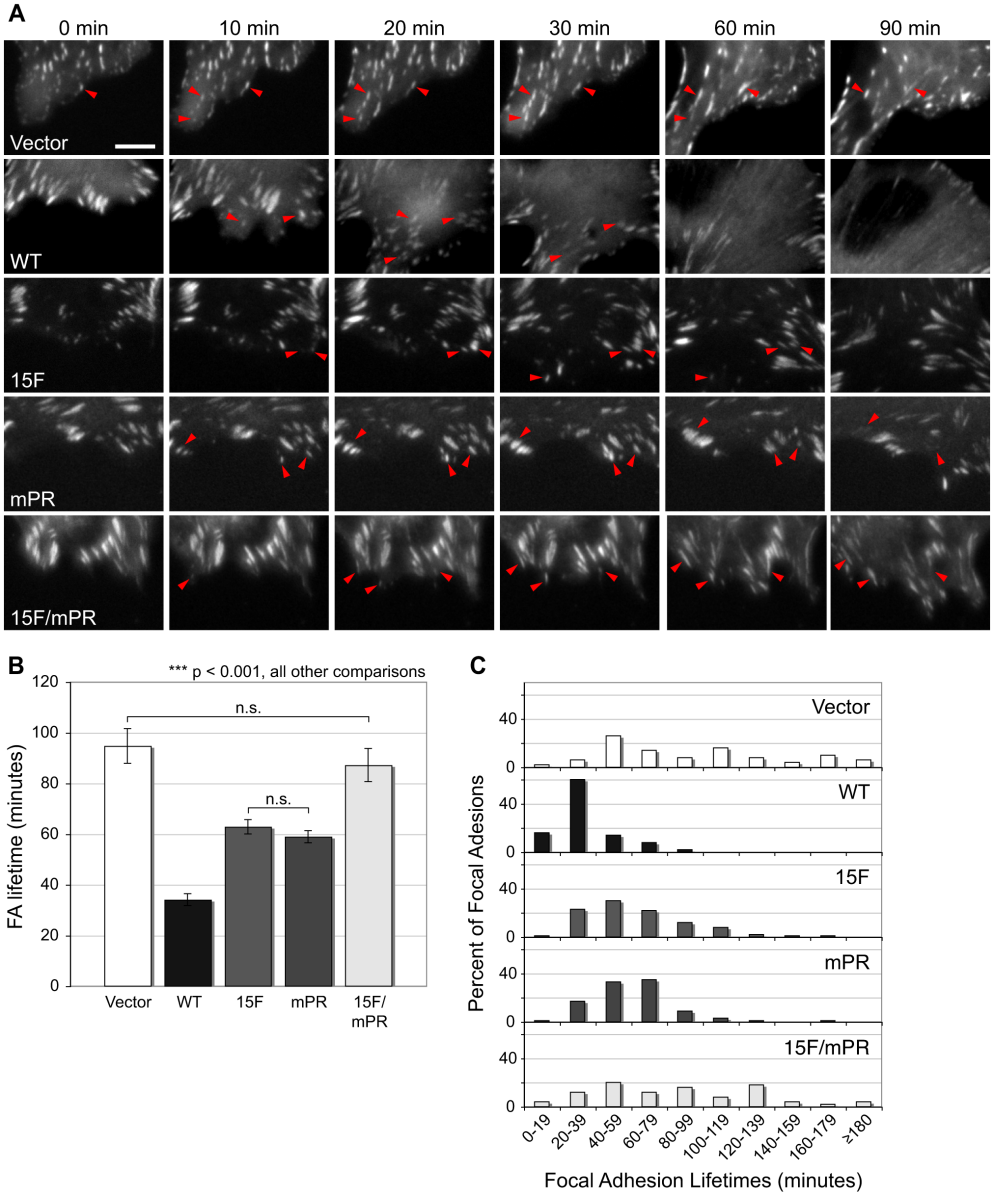
p130Cas SD and SBD signaling cooperate to shorten FA lifetimes. p130Cas WT and signaling deficient cells expressing a low level of mCherry-paxillin and migrating at the wound edge were viewed by wide field fluorescence microscopy with images captured at one-minute intervals. (A) Representative images at 0, 10, 20, 30, 60, and 90 minutes are shown. Arrowheads indicate FAs representative of mean values. Scale bar is 10 µm. Videos of the time sequences shown for p130Cas WT and 15F/mPR are presented in Supplemental Videos S1 and S2, respectively. (B) FA lifetimes determined by counting the number of sequential frames with mCherry-paxillin FA fluorescence intensity above background levels. Data shown represent the mean FA lifetimes of 50–100 FAs (10–20 FAs from five cells) for each cell type. Bars indicate s.e.m. Significance values were determined by one-way ANOVA followed by Tukey-Kramer post hoc testing; n.s. (not significant), ***p<0.001. (C) Histogram distributions of FA lifetimes, shown in 20-minute intervals. Note that the majority of WT FA lifetimes are less than 40 minutes while for all other cell types the majority of FAs lifetimes are greater than 40 minutes. Note also that the single mutants (15F and mPR) have similar distributions and that the Vector only control and 15F/mPR double mutant cells have similar distributions.

To evaluate how p130Cas signaling domains impact FA dynamics, mCherry-paxillin was transiently expressed in the various cell populations in order to mark FAs with a fluorophore, and then cells were visualized by time-lapse wide field fluorescence microscopy. Individual FAs forming at the leading edge of cells migrating into a wound were tracked from assembly through disassembly and their lifetimes were determined by counting sequential frames in which the adhesion signal was above background. From inspection of representative time-lapse images (Fig. 5A), it is apparent that most FAs in Vector cells are present throughout the 90-minute time-course whereas in WT cells many FAs are seen to form and fully disassemble after 30–40 minutes (Fig. 5A, arrowheads). FAs in the 15F/mPR cells also appear to persist throughout the 90-minute time-course, while FAs in both the 15F and mPR cells appear to have intermediate lifetimes. Quantitative analysis confirms that WT cells have the shortest mean FA lifetimes, Vector and 15F/mPR cells have the longest lifetimes, and 15F and mPR single mutant cells have intermediate lifetimes (Fig. 5B). The significant differences in mean FA lifetime among these groups reflect distinct distribution patterns such that the majority of FA lifetimes in WT cells fall under 40 minutes, those of 15F and mPR single mutant cells cluster between 20 and 80 minutes, and the FA lifetimes in Vector and 15F/mPR cells distribute even more broadly with no dominant peak (Fig. 5C). Similar to what we observed for cell migration rate, this additive defect indicates distinct roles for the SD and SBD in shortening FA lifetimes. To complement the analysis of FA dynamics, FA size distribution was determined from isolated cells stained for paxillin. Consistent with their shorter FA lifetimes, cells expressing WT p130Cas have a greater percentage of smaller FAs relative to cells expressing p130Cas signaling mutants (supplemental Fig. S2).

### P130Cas SBD-mediated SD signaling is implicated in enhancing rates of FA assembly and disassembly

**Figure 6:**
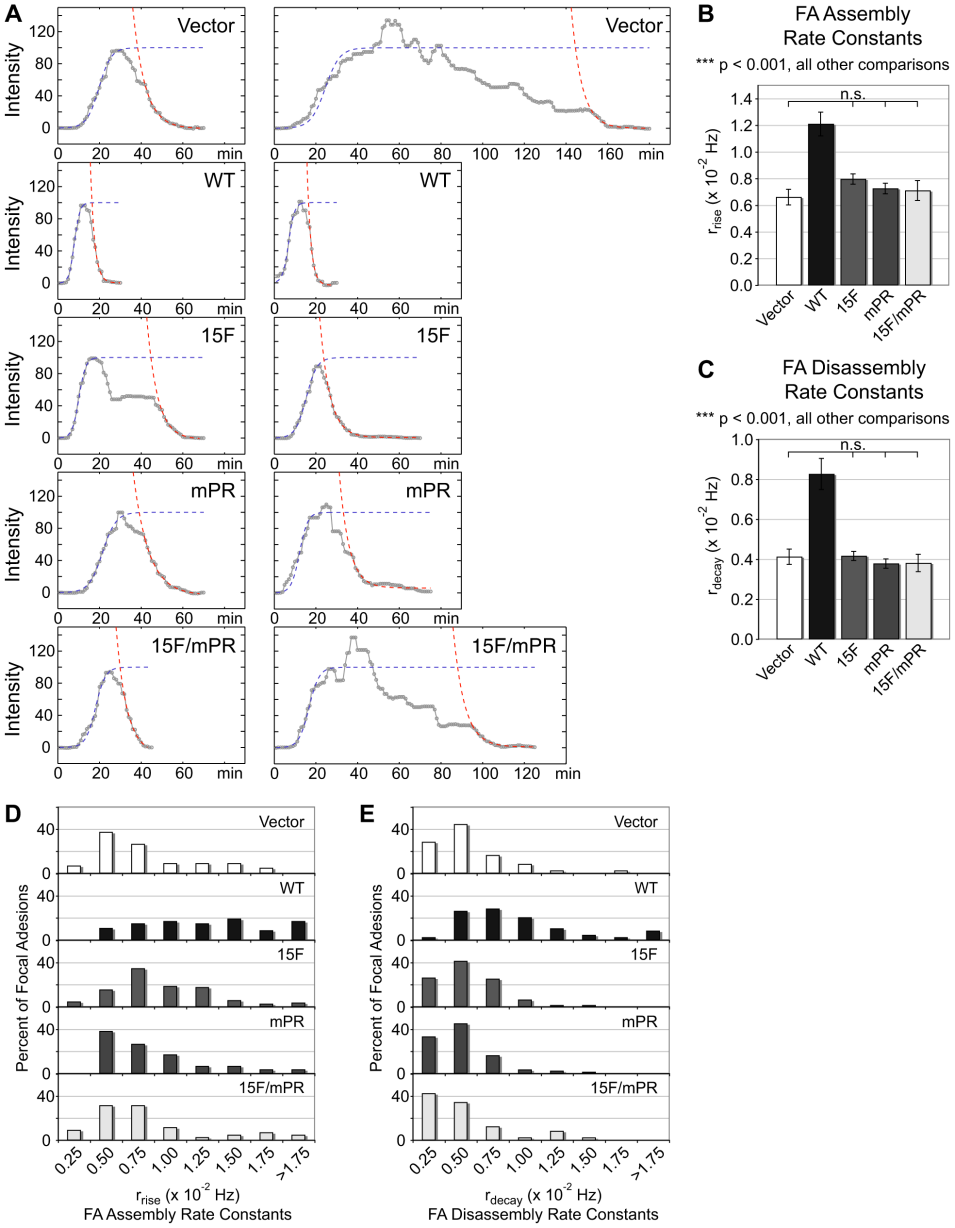
SBD-mediated SD signaling is implicated in stimulating both FA assembly and disassembly rates. Apparent rate constants of FA assembly (r_rise_) and disassembly (r_decay_) were determined by fitting FA assembly to a logistic function and the final FA disassembly to exponential decay. (A) Two examples of curve fits to FA intensity data (grey line) for assembly (purple dotted line) and disassembly (red dotted line) representative of the mean r_rise_ and r_decay_ are shown for each cell type. The graphs are normalized so that the modeled logistic function plateau equals 100. (B and C) Mean r_rise_ and r_decay_ values for each cell type are shown. Data represent the mean of 45–100 FAs from 5 cells. Bars indicate s.e.m. Significance values were determined by one-way ANOVA followed by Tukey-Kramer post hoc testing; n.s. (not significant), where all other comparisons are significant at ***p<0.001. (D and E) Histogram distributions of the r_rise_ and r_decay_ values for each cell type are shown in intervals of 0.25×10^−2^ Hz. In panel D, note that the majority of WT r_rise_ values are greater than 1.00×10^−2^ Hz while for all other cell types the majority of r_rise_ values are less than 1.00×10^−2^ Hz and have similar distributions. In panel E, note that the majority of WT r_decay_ values are greater than 0.50×10^−2^ Hz while for all other cell types the majority of r_rise_ values are less than 0.50×10^−2^ Hz and have similar distributions.

To better understand how p130Cas impacts FA dynamics, temporal fluorescence intensity profiles of the individual FAs evaluated in Fig. 5 were generated using a moving average of five data points to facilitate curve fitting. In the temporal profiles, increasing fluorescence intensity reflects FAs undergoing assembly, while decreasing intensity indicates disassembly. These profiles were then used to determine the rates of FA assembly and disassembly. Two representative fits of FA assembly and disassembly for each cell type are presented in Fig. 6A. FA assembly rate was modeled using a logistic function (exponential growth at early time points which plateaus to a maximum) and the final continuous phase of FA disassembly was modeled as simple exponential decay. This approach to determine apparent rate constants for FA assembly (r_rise_) and FA disassembly (r_decay_) follows on methodology for rate constant determination established by Webb et al. (2004), while using a greater percentage of the observed data to generate the rate constants.

Quantitative analysis showed that the mean FA assembly rate is significantly higher in p130Cas WT cells (1.5–1.8 fold) than for all other cell types (Fig. 6B). The mean FA assembly rates of Vector, 15F, mPR, and 15F/mPR cells were not significantly different from one another. Reflective of the mean differences, the majority of WT r_rise_ values are greater than 1.00×10^−2^ Hz, whereas the majority of r_rise_ values for all other cell types fall below this value (Fig. 6D). Similar results were obtained for measurements of FA disassembly. The mean FA disassembly rate is significantly higher in p130Cas WT cells (1.9–2.0 fold) than for all other cell types, and the mean FA disassembly rates are not significantly different between the other cells (Fig. 6C). Reflective of the mean differences, the majority of WT r_decay_ values are greater than 0.50×10^−2^ Hz, whereas the majority of r_decay_ values for all other cells fall below this value (Fig. 6E).

Similar to the results for leading edge protrusion, this analysis shows that the SD 15F mutation alone is sufficient to reduce both the FA assembly and disassembly rates to the levels seen in Vector cells. Once again, as SD tyrosine phosphorylation is impaired by the mPR mutation (Fig. 1C) [17], [19], the finding that 15F and mPR cells have similar r_rise_ and r_decay_ values indicates that the primary signaling role of the SBD in enhancing the rates of FA assembly/disassembly is by mediating SD tyrosine phosphorylation.

### P130Cas SD and SBD signaling domains have distinct roles in sustaining FA disassembly

**Figure 7:**
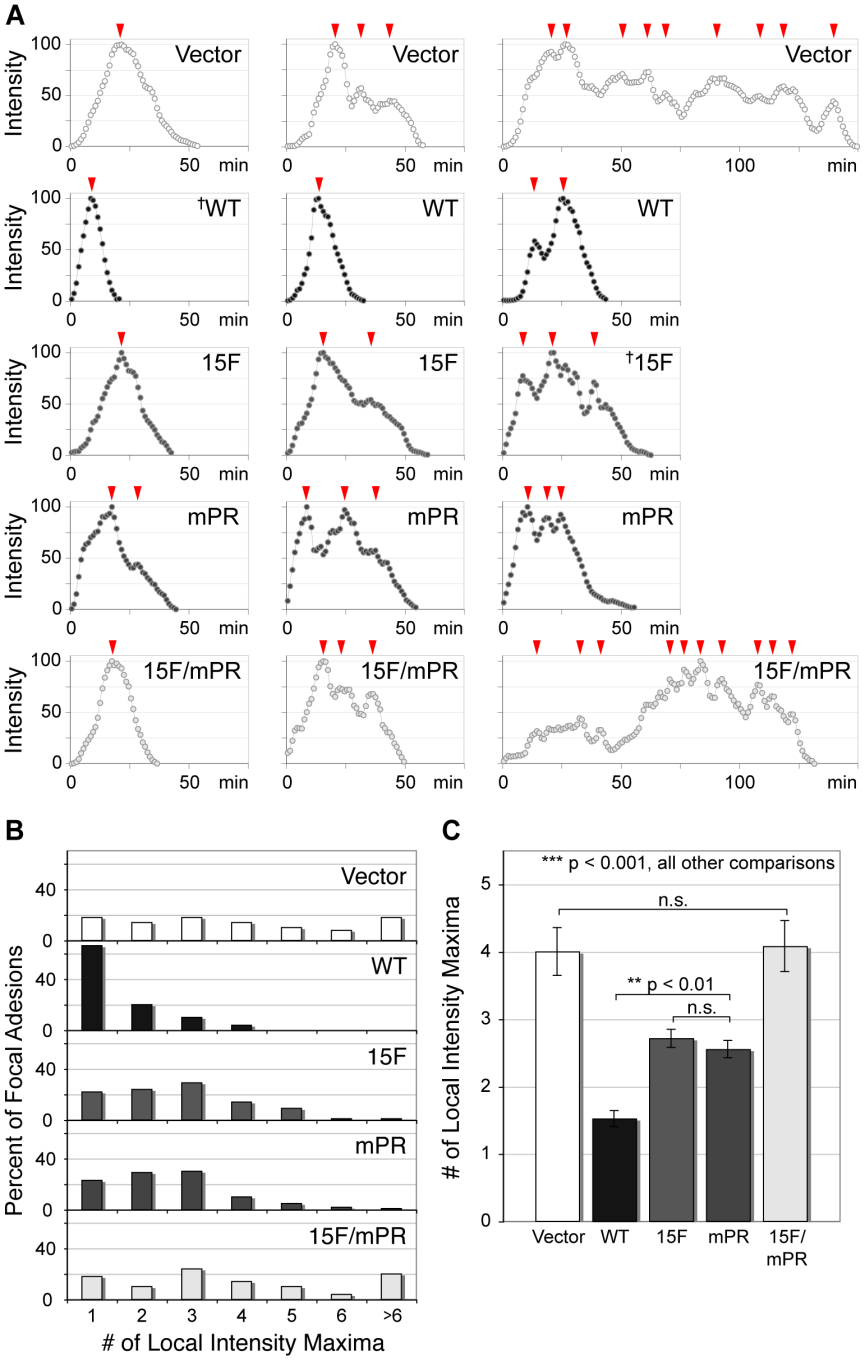
FA profile complexity is increased in the absence of p130Cas SD and SBD signaling. FA intensity profiles were created from time-lapse images. (A) Three FA intensity profiles representative of each cell type are shown, normalized with the maximum intensity at 100. Arrowheads indicate local intensity maxima. † indicates intensity profiles shown in supplemental Fig. S3 with corresponding kymographs. (B) Histogram distributions of the number of local maxima per FA intensity profile for each cell type. The majority (66%) of WT FAs have a single maximum. The single mutants are evenly distributed between 1–3 local maxima. The Vector and the 15F/mPR mutant cells show a broad distribution with a substantial number of FAs with >5 local intensity maxima. (C) FA profile complexities for each cell type were quantified by counting the number of local intensity maxima. Data shown represent the mean number of local maxima for 50–100 FAs (10–20 FAs from each of five cells). Bars indicate s.e.m. Significance values were determined by one-way ANOVA followed by Tukey-Kramer post hoc testing; n.s. (not significant), **p<0.01, ***p<0.001.

In modeling FA disassembly rates, we noted prominent discontinuous FA disassembly, particularly in the p130Cas signaling deficient cells. In order to quantify this phenomenon, we re-plotted the temporal intensity profiles using a moving average of 3 data points to highlight the numerous intensity peaks. From representative profiles (Fig. 7A), it can be seen that FAs with shorter lifetimes have simple bell-shaped profiles with a single peak of intensity while longer-lived FAs have more complex profiles with numerous local intensity maxima (arrowheads). The multiple local intensity maxima are indicative of abortive disassembly attempts. Quantitative analysis (Figs. 7B and 7C) showed that FAs in p130Cas WT cells mostly have a single intensity maximum while FAs from the cells deficient in p130Cas signaling tend to have more complex profiles. For 15F and mPR cells, 2–3 local intensity maxima are most common. For Vector and 15F/mPR cells, there is an even broader distribution of FA local intensity maxima number with >5 often observed (Fig. 7B). We note that FAs with multiple intensity maxima typically exhibit sliding behavior. This can be readily seen from a representative kymograph shown in Supplemental Fig. S3B. The mean FA local intensity maxima number is significantly lower in the WT cells compared to all other cell types, while 15F and mPR cells have significantly fewer local intensity maxima than the Vector and 15F/mPR cells. These results indicate that signaling deficiencies in the SD and SBD of p130Cas extend FA lifetimes primarily due to a failure to sustain FA disassembly.

As was observed for cell migration rate, the additive effect of the 15F and mPR signaling variants indicates that sustained FA disassembly is regulated by distinct p130Cas signaling functions of the SD and SBD.

## Discussion

**Figure 8:**
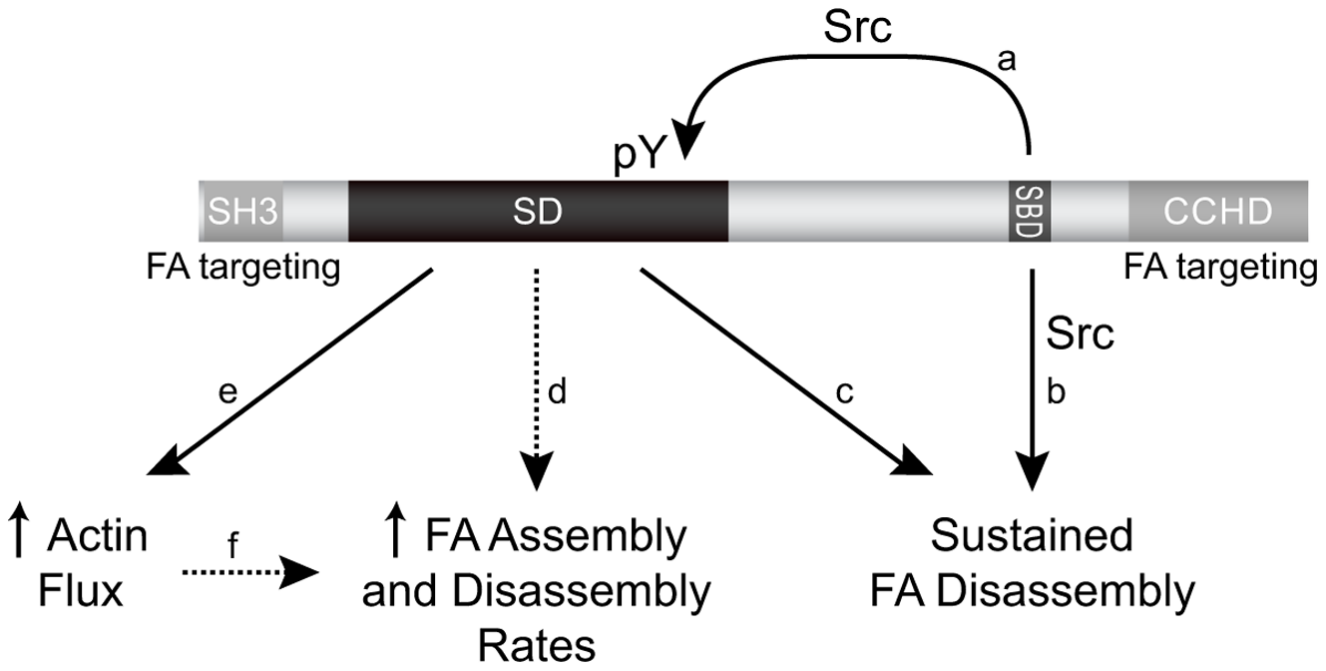
Summary of p130Cas SD and SBD signaling in the regulation of FA and actin dynamics. The SBD activates Src to promote SD tyrosine phosphorylation (a) and also promotes sustained FA disassembly through a distinct pathway (b). SD tyrosine phosphorylation acts to sustain FA disassembly (c), stimulate the rates of FA assembly and disassembly (d), and promote leading edge actin flux (e). Increased FA assembly/disassembly rates may be a direct effect of SD signaling on adhesion dynamics (d) or an indirect effect resulting from leading edge protrusion driving more rapid FA assembly/disassembly (e, f). Dotted lines indicated that the effect of SD on FA assembly and disassembly rates could be either direct or indirect.

We used live cell imaging techniques to investigate the extent to which p130Cas signaling domains contribute to the dynamic adhesion and membrane protrusion events that drive cell motility. These properties were examined in a monolayer wound healing response, comparing MEFs expressing WT p130Cas versus variants in which the signaling capacities of the SD and/or the SBD were disrupted by point mutations. Our results (summarized in Fig. 8) indicate that both the tyrosine phosphorylation sites in the SD and the Src SH3 binding site in the SBD contribute significantly to the ability of p130Cas to stimulate cell migration through enhancing FA and actin dynamics. Loss of either signaling function fully impaired p130Cas-enhanced rates of plasma membrane protrusion and FA assembly/disassembly. However, the ability of p130Cas to enhance cell migration rate and maintain uninterrupted FA disassembly were fully abolished only when the SD and SBD functions were simultaneously impaired. These findings indicate that the p130Cas SBD has a function in maintaining FA disassembly and promoting cell migration that is distinct from its capacity to recruit and activate Src to promote SD tyrosine phosphorylation.

Neither the 15F mutation (loss of tyrosine phosphorylation sites) in the SD nor the mPR mutation (disruption of the Src SH3-binding motif) in the SBD fully impaired the ability of p130Cas to promote cell migration. But combining both mutations (15F/mPR) fully abolished the migration-promoting activity of p130Cas (Fig. 2). In contrast to our results, another study reported that mutation of either the SD or the SH3 binding motif of the SBD was sufficient to fully impair p130Cas-enhanced cell migration [26] and thus a distinct role for the SBD could not be attributed. Our finding of a distinct role for the SBD in cell migration is consistent with the multiple biochemical functions attributed to this domain [16], [27], [28].

Upon tyrosine phosphorylation, the p130Cas SD interacts with the SH2/SH3 adapter proteins, Crk and Nck, and thus may recruit these activators of actin assembly to stimulate plasma membrane protrusion [13], [14], [24]. Therefore we addressed whether p130Cas-enhanced cell migration could be attributed to an enhanced rate of membrane protrusion at the leading edge. We found that both the SD and SBD are required to promote leading edge protrusion. Cells expressing p130Cas WT had approximately 30% higher protrusion rates relative to Vector control cells and to all three p130Cas mutant cells (15F, mPR, and 15F/mPR). Thus tyrosine phosphorylation, likely mediated by Src recruitment to the SBD, is the key p130Cas signaling event driving enhanced protrusion. The data do not indicate a distinct role for the SBD in driving protrusion and thus do not fully account for the p130Cas mediated cell migration response. In extending this analysis we demonstrated that WT p130Cas stimulated actin assembly and retrograde flow at the leading edge lamellipodium and that these effects were fully blocked by the SD 15F mutation. Thus new experimental evidence linking p130Cas signaling to leading edge actin dynamics was provided.

Observations that p130Cas SD tyrosine phosphorylation occurs primarily within mature FAs located away from the leading edge [19], [22] suggest p130Cas signaling may act to regulate FA dynamics during cell migration. Indeed, Cas −/− MEFs are reported to have a lower rate of FA disassembly compared to cells expressing p130Cas [25] but which, if any, of the p130Cas signaling functions are involved in this process was not previously addressed. We found that both the SD tyrosine phosphorylation sites and the SBD Src SH3-binding motif have important roles in regulating FA dynamics. Approximately 2-fold higher rates of FA assembly and disassembly were observed in p130Cas WT cells relative to Vector control cells and to all p130Cas mutant cells (15F, mPR, and 15F/mPR). The similar defects observed in all p130Cas mutants implicate SD tyrosine phosphorylation mediated by Src recruitment to the SBD as the key signaling function of p130Cas in stimulating FA assembly and disassembly rates, similar to leading edge protrusion. That these two processes show similar dependencies on p130Cas signaling domains suggests they may be coupled as has been speculated [3].

We found that FAs from cells deficient in p130Cas SD and SBD signaling functions (15F/mPR and Vector) have ∼3-fold longer lifetimes in comparison to p130Cas WT cells (Fig. 5). This cannot be fully attributed to the differences in assembly/disassembly rate constants. Rather, the defect of extended FA lifetimes was linked to unusual FA temporal fluorescence intensity profiles containing multiple peaks (Fig. 7). Such profiles are indicative of a defect in sustaining FA disassembly. FAs with multiple intensity maxima typically exhibit sliding behavior as is evident from the kymography data shown in Fig. S3. It is notable that the full defects of extended FA lifetime and abortive FA disassembly were achieved only when both the SD and SBD mutations were combined, similar to our findings on cell migration rate. Thus we conclude that a major function of p130Cas in promoting cell migration is to sustain FA disassembly, with SD tyrosine phosphorylation and SBD SH3-binding motifs having distinct roles in this process.

It is interesting to speculate that p130Cas signaling may sustain FA disassembly as a consequence of its capacity to stimulate localized actin flux, in this case originating from mature FAs under high contractile tension. The coupled retrograde movement of actin and FA components has been likened to a ‘slippage clutch” whereby cells transduce mechanical signals and transmit force to the ECM [29], [30], [31]. Moreover p130Cas in FAs may act as a “mechanotransducer” whereby stress-fiber generated tension triggers a conformational change that enhances Src-mediated SD tyrosine phosphorylation [21]. The resultant signaling by p130Cas may thus act to stimulate local actin assembly at the stress fiber/FA interface, thereby disengaging the slippage clutch to release tension and allow FA disassembly to occur. In this model, the aborted FA disassemblies observed in p130Cas signaling deficient cells would be due to repeated failures to fully disengage the clutch. A possible role for p130Cas SD signaling promoting actin assembly in mature FAs was suggested by another recent study from our group where Src activity was specifically elevated in FAs through expression of a FAK-Src chimeric protein [32]. This resulted in greatly elevated p130Cas SD tyrosine phosphorylation and a striking display of transient actin assembly in mature FAs. Results from our present study further indicate that the p130Cas SBD has a role in sustaining FA disassembly that is distinct from that of the SD. In this regard, we note that SBD-activated Src can phosphorylate other substrates including paxillin and cortactin [16], both of which have been implicated in regulating FA dynamics [25], [33], [34], [35]. Interestingly, a cortactin mutant that cannot undergo Src phosphorylation extends FA disassembly time and appears to give rise to FA profiles with multiple intensity peaks [34], similar to what we observed in p130Cas signaling deficient cells. It will be of interest for future studies to evaluate the role of p130Cas-activated Src substrates in FA dynamics.

## Materials and Methods

### Cells and cell culture

Cas −/− and Cas +/+ MEFs [11] were kindly provided by Hisamaru Hirai (University of Tokyo). MEFs were maintained in Dulbecco's Modified Eagle's Medium (Mediatech, Manassas, VA) supplemented with 10% fetal bovine serum (Atlanta Biologicals, Lawrenceville, GA), 1% antibiotic/antimicotic (Mediatech, Manassas, VA), and 1% non-essential amino acids (Invitrogen, Carlsbad, CA). The retroviral packaging cell line Phoenix Ectotropic (E) kindly provided by Gary Nolan (Stanford University), was maintained as previously described [36].

### Antibodies

Monoclonal antibody against total p130Cas was obtained from BD Transduction Laboratories (San Jose, CA). Mouse monoclonal antibody against actin was obtained from Sigma (St. Louis, MO). The phosphospecific pCas antibodies raised against p130Cas SD tyrosines 165, 249, and 410 were from Cell Signaling Technology (Beverly, MA). Horseradish peroxidase conjugated goat-anti-mouse IgG and goat-anti-rabbit secondary antibodies were obtained from Jackson ImmunoResearch Laboratories, Inc. (West Grove, PA).

### Plasmids and protein expression

The LZRS-MS-IRES-GFP retroviral vector [37] was used to express p130Cas variants in conjunction with cytoplasmic GFP from a bicistronic transcript. The cell populations used for this study were obtained by three sequential rounds of viral infection of Cas −/− MEFs followed by one round of fluorescence-activated cell sorting to select cells with low levels of GFP expression. P130Cas wild type (WT) and mutational variants 15F, mPR, and 15F/mPR were expressed in Cas −/− MEFs. The expression constructs for p130Cas WT and 15F [15] and mPR [19] have been previously described. The 15F/mPR variant was generated for this study using standard molecular biology techniques. All p130Cas variants were verified by sequencing.

Two plasmids were used for transient expression. mCherry-C1-paxillin (previously described [12], [38]) and mCherry-Actin. Actin was subcloned from Clontech pEGFP-actin using NheI and BglII restriction sites into the mCherry-C1 plasmid (provided by Maria Nemethova, Vienna, Austria). The resulting product is expressed with an N-terminal mCherry tag. Cells were transfected for transient expression using Lipofectamine 2000 (Invitrogen, Carlsbad, CA).

### Immunoblotting

Subconfluent cell cultures were washed with phosphate buffered saline (PBS) and lysed in modified RIPA buffer [36]. Whole cell lysates were separated on 7% SDS-polyacrylamide gels and transferred onto nitrocellulose membrane. Nonspecific activity was blocked by incubating 1 hour at room temperature in Tris-buffered saline containing 3% nonfat dry milk. Membranes were incubated overnight at 4°C in primary antibody, washed extensively with TBS-T (0.05% Tween-20), and then incubated 1 hour at room temperature with secondary antibody. Immunoblots were visualized by enhanced chemiluminescence.

### Immunoprecipitation

Subconfluent cell cultures were washed with PBS and lysed in 1% SDS boiling buffer; NP-40 buffer (50 mM This-Cl, pH 7.4, 150 mM NaCl, 5 mM EDTA, 50 mM NaF, 1% Nonidet P-40, 1 µg/ml Aprotinin, 100 µM Leupeptin, 1 mM Benzamidine, 100 µM sodium orthovanadate, 50 µg/ml PMSF) plus 1% SDS. Samples were heated to 100°C for 5 minutes, then diluted with NP-40 buffer to 0.1% SDS. For immunoprecipitation, 1000 µg of total protein in 800 µl of lysis buffer was incubated with 2 µg CAS-TL antibody, and protein was recovered on protein G-Sepharose 4B (Sigma-Aldrich, St Louis, MO). Immunoprecipitates were then immunoblotted as described.

### Wound healing migration assay

Cells were grown to confluence on glass coverslips coated with 1 µg/ml fibronectin (from human plasma, Sigma-Aldrich, St Louis, MO or Calbiochem, San Diego, CA). A pipette tip was used to make a scratch wound, then the cells were transferred to media supplemented with 10 mM HEPES (Mediatech, Manassas, VA) and mounted in a heated chamber (Warner Instruments, Hamden, CT) to maintain cells at 37°C. Cells migrating into the denuded area were visualized on a Nikon Eclipse TE2000-E2 inverted microscope equipped with a Perfect Focus System using a 20× Plan Apo DIC objective lens. Using IPlab software (Scanalytics, Fairfax, VA), frames were captured on a CoolSnapHQ camera (Photometrics, Tucson, AZ) every five minutes for 8 hours. To determine total migration distance for each cell population, nuclei of seventy-five cells (25 each from three independent experiments) migrating at the wound edge were tracked using the ImageJ (National Institutes of Health, Bethesda, MD) Manual Tracking plugin. The mean velocity (microns/hour) was calculated for each cell population over six hours (two to eight hours post wounding). Any cells that divided during the movie were excluded from the analysis.

### Leading edge DIC kymography

Cells were prepared as in the wound healing migration assay and DIC images were captured using a 60× Plan Apo 1.4 NA oil-immersion DIC objective lens. Frames were captured every 3 seconds for 15 minutes starting at an average of 4.6 hours (s.d. +/− 0.5 hours) post wounding for all cells. Three kymographs were generated for each cell. The time and distance of all protrusions that were parallel to the line of analysis during the 15-minute period were used to calculate the mean protrusion velocity. The total protrusion distance and time for all three kymographs were summed to generate a mean protrusion velocity for each cell. 60–90 cells (15 each from 4–6 separate wound assays) were analyzed for each cell type.

### Live cell fluorescence imaging

Cells were transiently transfected with mCherry-C1-paxillin or mCherry-C1-actin using Lipofectamine 2000, then grown to confluence (48 hours) on 1 µg/ml fibronectin-coated coverslips. Wounds were generated and cells mounted as in the wound healing migration assay. Cells migrating at the wound edge and expressing a low, but detectable level of fluorescently-tagged protein were selected for imaging. Cells expressing mCherry-tagged proteins were visualized using a Pinkel triple filter set (Semrock, Rochester, NY) and a TIRF 100× 1.49 NA oil-immersion lens. Images were captured on a back-illuminated EM-CCD camera, Cascade 512B (Photometrics).

Wide field fluorescence images of FA dynamics were captured every 60 seconds for 4 hours starting at an average of 2.5 hours post wounding (s.d. +/− 0.5 hours) for all cells. Photobleaching of leading edge mCherry-actin was achieved using a 10 mW DPSS laser 85YCA010 (Melles Griot, Albuquerque, NM) and a custom-made lens (Nikon) in the position of the filter cube to focus the laser in the focal plane. A pre-bleach image was captured, the leading edge actin was photobleached for 15 seconds, then images were captured every second for 60–90 seconds. Cells expressing mCherry-actin were imaged at an average of 5.1 hours post wounding (s.d. +/− 0.9 hours) for all cells.

### Leading edge wide field fluorescence kymography

Actin dynamics were quantified from mCherry-actin kymographs (see Fig. 4C). A photobleached region of mCherry-actin at the leading edge was used as a reference point (similar to [39], [40]). All three rates were calculated over the same time period immediately after photobleaching. The rate of leading edge protrusion was determined by tracking the forward movement of the newly incorporated fluorescent actin. The rate of retrograde flow was determined by tracking the rearward movement of the sharp boundary of newly incorporated fluorescent actin after bleaching [39], [40]. The rate of actin assembly was calculated as the sum of the absolute values of the retrograde flow and leading edge protrusion rates. The calculation is illustrated in a schematic in Fig. 4C.

### FA lifetimes

The lifetimes of individual FAs forming at the leading edge (50–100 FAs, 10–20 each from 5 cells) of cells migrating at a wound edge were quantified by tracking FAs from assembly through disassembly and counting the number of sequential frames in which individual FAs had signal greater than background levels.

### FA intensity profiles

Using ImageJ, FAs were outlined in each frame of the FA time-course. Background subtracted total pixel intensity was measured from each frame, using immediately adjacent pixels (not in the FA) to measure background intensity. To reduce noise, a moving average of either five or three consecutive intensity data points of background-subtracted FA intensity measurements was plotted (used for curve-fitting and local maxima quantification, respectively).

### FA assembly and disassembly rate calculations

To reduce noise and facilitate curve fitting, temporal intensity profiles with a moving average of five consecutive intensity data points of background subtracted FA intensity measurements were used. Each data set was fit for six parameters using a least squares fitting routine in Matlab, three parameters for FA assembly, initial growth to maximum signal (r_rise_, I_0_, I_lim_) and three for FA disassembly, final decay (r_decay_, A, I_∞_). The assembly was fit to a logistic function:
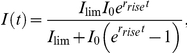
where I = signal intensity, t = time, I_0_ = signal intensity at t = 0, r_rise_ = rate of exponential growth during the initial rise at small I values, and I_lim_ = signal as t→∞, also called the limiting value. This function is fit using a portion of the data, from the first non-zero point to one point beyond the maximum. Three additional parameters are determined by fitting the final disassembly to a simple exponential decay plus offset:
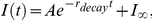
where A is the amplitude of the decay, r_decay_ is the rate of decay, and P_∞_ is the offset from zero signal. The range of data employed in this fitting was selected in order to capture only the final decay, while fitting to as much signal as possible for each intensity data set. This range of data varies with each data set, but begins at a point with an average value of 51% (s.d. ± 22%) of the fitted limiting value (I_lim_) and continues until the FA intensity is indistinguishable from the background. For statistical analysis of FA assembly, poor fits (extreme values greater than two standard deviations above the mean or fits that used fewer than five data points in the exponential rise phase to approximate FA growth) were eliminated from the analysis.

### Local maxima quantification

To reduce noise, a moving average three consecutive intensity data points of background-subtracted FA intensity measurements was plotted. The data were normalized so that the maximum intensity value for each FA equaled 100 (arbitrary units), and the number of local maxima in each intensity profile was quantified. A local maximum was defined as at least three consecutive rising points or an increase of ∼10% of the maximum intensity value followed by at least three consecutive falling points or a decrease of ∼10% of the maximum intensity value.

### Statistical analysis

To compare the means of the numerous groups, one-way analysis of variance (ANOVA) followed by the Tukey-Kramer post hoc test was used to determine significance for pair-wise comparisons of all samples. Values were considered not significant (n.s.) when p≥0.05.

Additional information on Materials and Methods pertaining to supplemental figures is available as Materials and Methods S1.

## Supporting Information

Figure S1 p130Cas SD and SBD signaling facilitate the initiation of cell spreading but have no effect on the final spread cell area. Cells were plated onto glass coverslips coated with 1 µg/ml fibronectin and imaged for 2 hours by live DIC microscopy as they attached and spread. (A) Histogram distributions of the time at which cells initiated spreading after replating, and the number of cells that did not spread (DNS) at the 2-hour time point. A total of 211–298 cells for each cell type from 3 separate replating assays were analyzed. Cells that divided during the two-hour period were excluded from the analysis. (B) The mean time of spreading initiation was quantified from the cells that spread within the first two hours. Data shown represent the mean spreading initiation times determined from 145–256 cells for each cell type. Bars indicate s.e.m. (C) Box and whisker plots of spread cell area for cells that were fully spread at two hours after replating. The area of 74–93 cells for each cell type from 3 separate replating assays was measured. The (+) indicates the median, the bottom and top of the box indicate the 25th and 75th percentiles, and the lower and upper whiskers indicate the 10th and 90th percentiles. Significance values were determined by one-way ANOVA followed by Tukey-Kramer post hoc testing; n.s. (not significant), *p<0.05.
(0.21 MB TIF)

Figure S2 Defects in p130Cas SD and SBD signaling give rise to larger FAs in the front of polarized cells. FA size was assessed by wide field fluorescence imaging of fixed cells immunostained for paxillin to mark FAs. Single cells with a clear polarized morphology were selected and a mask of the front FAs was generated from the paxillin image. (A–B) Representative fixed images stained for paxillin, FA masks, and FA distribution pattern are shown for a Vector and WT cell. Scale bars are 10 µm. (C) Mean front FA size was quantified by evaluating FAs from 10–11 cells for each cell type. Bars indicate s.e.m. Significance values were determined by one-way ANOVA followed by Tukey-Kramer post hoc testing; n.s. (not significant), **p<0.01.
(0.53 MB TIF)

Figure S3 Kymographs of representative FAs showing single versus multiple intensity peaks. To visually illustrate the FA dynamics represented by peaks in the intensity profiles, two representative FA kymographs were generated corresponding to intensity profiles in Fig 7A, that have either a single peak (A) or multiple peaks (B). The images on the left show the FAs at the indicated time point with arrows designating both the region used for the kymograph and the direction of migration. Kymographs are shown on the right, presented above the corresponding intensity profiles. Red arrowheads indicate local intensity maxima, as in Fig 7A. Scale bars are 2 µm.
(0.15 MB TIF)

Materials and Methods S1 (0.05 MB DOC)

Video S1 FA dynamics in a representative p130Cas WT cell. Video corresponding to the p130Cas WT time series presented in Fig 5 (120 frames captured at 60-second intervals and presented at 10 frames per second).
(0.64 MB MOV)

Video S2 FA dynamics in a representative p130Cas 15F/mPR cell. Video corresponding to the p130Cas 15F/mPR time series presented in Fig 5 (120 frames captured at 60-second intervals and presented at 10 frames per second).
(0.73 MB MOV)
