# Supplementary figures and images for: P130Cas Src-Binding and Substrate Domains Have Distinct Roles in Sustaining Focal Adhesion Disassembly and Promoting Cell Migration
Source: PLoS One. 2010 Oct 18;5(10):e13412. doi: 10.1371/journal.pone.0013412 (PMC2956669; doi:10.1371/journal.pone.0013412)

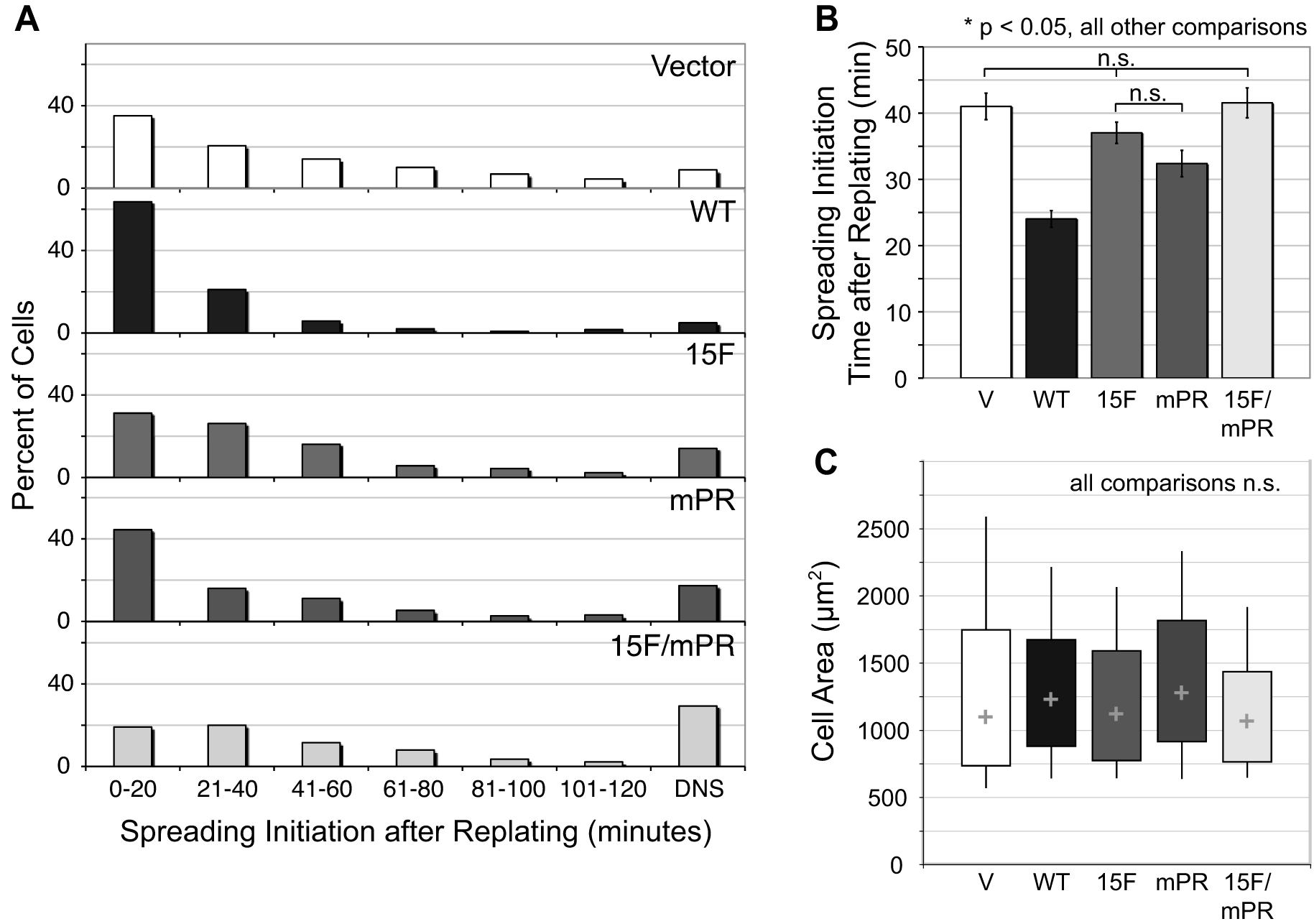

Supplement: Figure S1 — p130Cas SD and SBD signaling facilitate the initiation of cell spreading but have no effect on the final spread cell area. Cells were plated onto glass coverslips coated with 1 µg/ml fibronectin and imaged for 2 hours by live DIC microscopy as they attached and spread. (A) Histogram distributions of the time at which cells initiated spreading after replating, and the number of cells that did not spread (DNS) at the 2-hour time point. A total of 211–298 cells for each cell type from 3 separate replating assays were analyzed. Cells that divided during the two-hour period were excluded from the analysis. (B) The mean time of spreading initiation was quantified from the cells that spread within the first two hours. Data shown represent the mean spreading initiation times determined from 145–256 cells for each cell type. Bars indicate s.e.m. (C) Box and whisker plots of spread cell area for cells that were fully spread at two hours after replating. The area of 74–93 cells for each cell type from 3 separate replating assays was measured. The (+) indicates the median, the bottom and top of the box indicate the 25th and 75th percentiles, and the lower and upper whiskers indicate the 10th and 90th percentiles. Significance values were determined by one-way ANOVA followed by Tukey-Kramer post hoc testing; n.s. (not significant), *p<0.05. (0.21 MB TIF) [file pone.0013412.s001.tif]

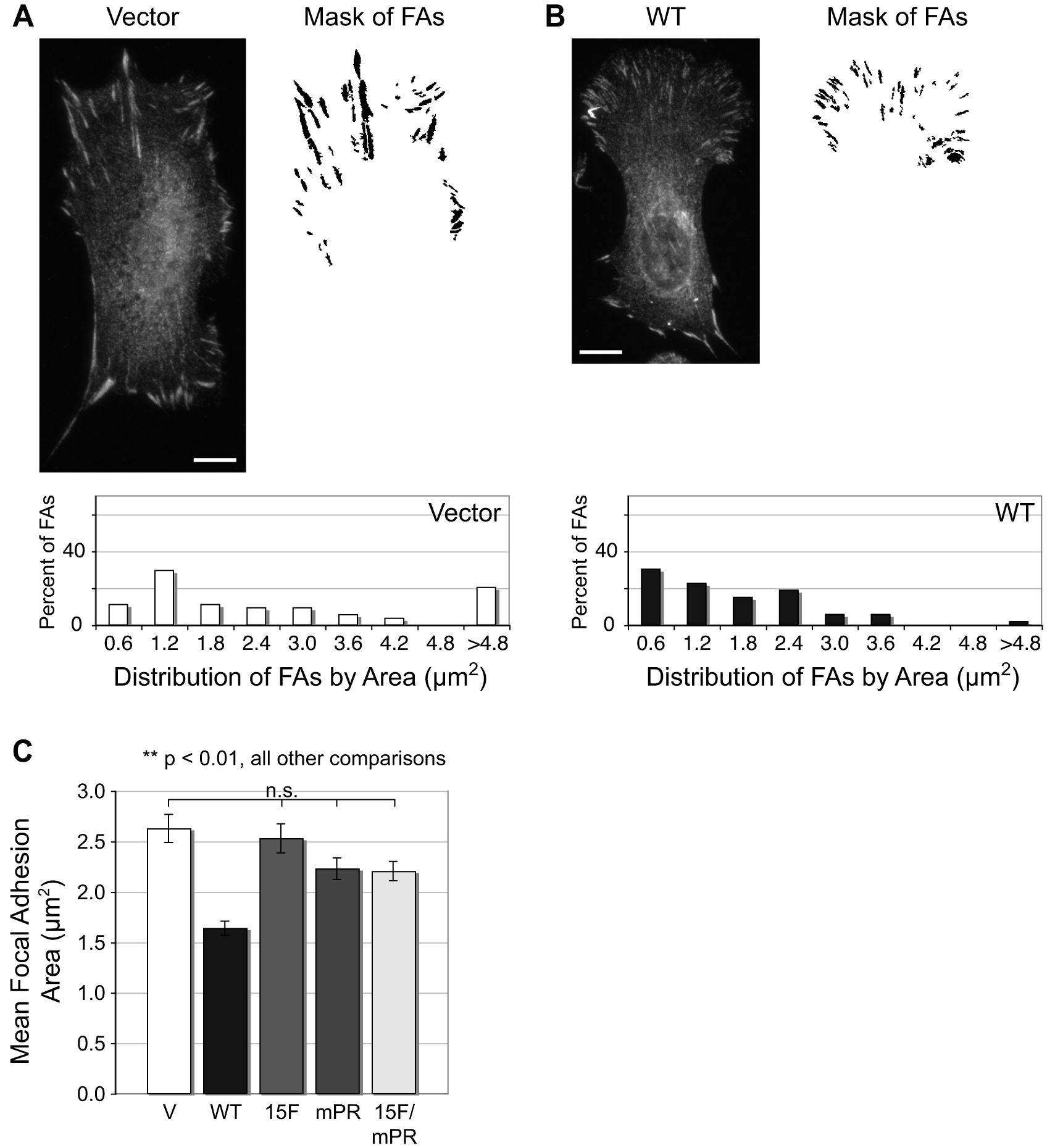

Supplement: Figure S2 — Defects in p130Cas SD and SBD signaling give rise to larger FAs in the front of polarized cells. FA size was assessed by wide field fluorescence imaging of fixed cells immunostained for paxillin to mark FAs. Single cells with a clear polarized morphology were selected and a mask of the front FAs was generated from the paxillin image. (A–B) Representative fixed images stained for paxillin, FA masks, and FA distribution pattern are shown for a Vector and WT cell. Scale bars are 10 µm. (C) Mean front FA size was quantified by evaluating FAs from 10–11 cells for each cell type. Bars indicate s.e.m. Significance values were determined by one-way ANOVA followed by Tukey-Kramer post hoc testing; n.s. (not significant), **p<0.01. (0.53 MB TIF) [file pone.0013412.s002.tif]

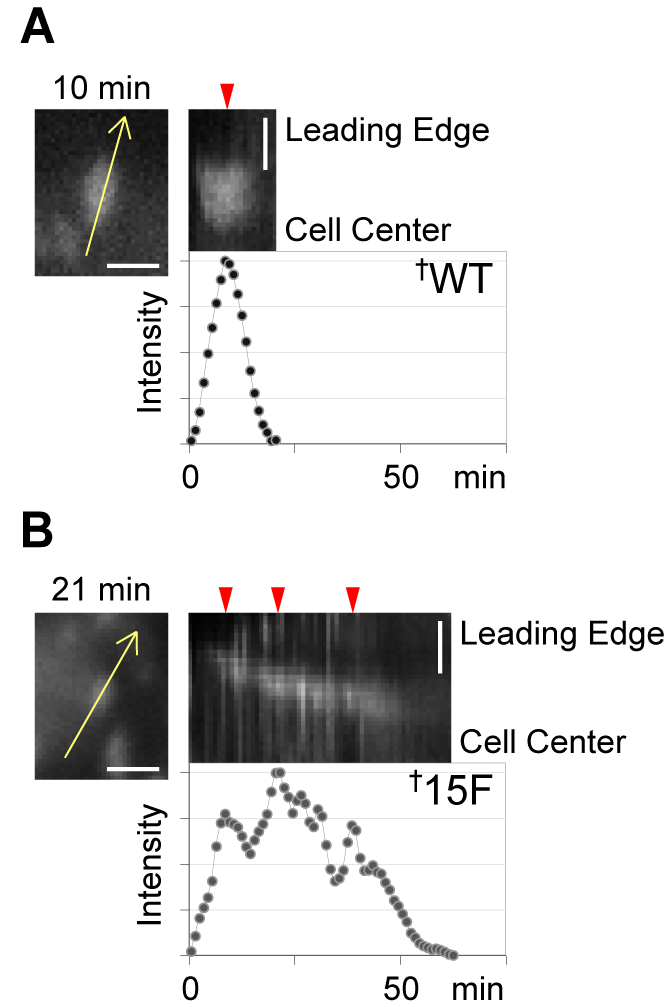

Supplement: Figure S3 — Kymographs of representative FAs showing single versus multiple intensity peaks. To visually illustrate the FA dynamics represented by peaks in the intensity profiles, two representative FA kymographs were generated corresponding to intensity profiles in Fig 7A, that have either a single peak (A) or multiple peaks (B). The images on the left show the FAs at the indicated time point with arrows designating both the region used for the kymograph and the direction of migration. Kymographs are shown on the right, presented above the corresponding intensity profiles. Red arrowheads indicate local intensity maxima, as in Fig 7A. Scale bars are 2 µm. (0.15 MB TIF) [file pone.0013412.s003.tif]
