# Supplementary material for: P130Cas Src-Binding and Substrate Domains Have Distinct Roles in Sustaining Focal Adhesion Disassembly and Promoting Cell Migration
Source: PLoS One. 2010 Oct 18;5(10):e13412. doi: 10.1371/journal.pone.0013412 (PMC2956669; doi:10.1371/journal.pone.0013412)
Supplement: Materials and Methods S1 — (0.05 MB DOC) [file pone.0013412.s004.doc]

P130Cas Src-Binding and Substrate Domains Have Distinct Roles in Sustaining Focal Adhesion Disassembly and Promoting Cell Migration

Leslie M. Meenderink^1^, Larisa M. Ryzhova^1^, Dominique M. Donato^1^, Daniel F. Gochberg^2,3,4^, Irina Kaverina^1^, Steven K. Hanks^1*^

^1^ Department of Cell and Developmental Biology, Vanderbilt University School of Medicine, Nashville, TN 37232

^2^ Department of Radiology and Radiological Sciences, Vanderbilt University School of Medicine, Nashville, TN 37232

^3^ Department of Physics and Astronomy, Vanderbilt University, Nashville, TN 37232

^4^ Vanderbilt University Institute of Imaging Science (VUIIS), Nashville, TN 37232

* Corresponding Author

**Materials and Methods for Supplemental Materials**

**Antibodies**

Monoclonal antibody against paxillin was obtained from BD Transduction Laboratories (San Jose, CA). Cy3-conjugated AffiniPure donkey anti-mouse IgG secondary antibody was obtained from Jackson ImmunoResearch Laboratories Inc. (West Grove, PA).

**Cell Spreading Assay**

Subconfluent cells were plated onto coverslips coated with 1 µg/ml fibronectin and immediately mounted in a heated chamber (Warner Instruments, Hamden, CT) to maintain cells at 37˚C and imaged as they attached and spread. Images were captured every 5 minutes for 2 hours using a 20x Plan Apo DIC objective lens. Cells were scored as having initiated spreading after obvious protrusions had emanated from the cell periphery. Cells that divided during the analysis were not scored. Cells that attached, but neither initiated spreading nor divided during the 2-hour period were scored as DNS (did not spread). Spread cell area was quantified by analyzing cells that fully spread, and then measuring the cell area at 2-hours after plating. Cells that were still rounded or had just initiated spreading were not included in the cell area analysis.

**Immunostaining**

Cells growing at low density were plated and allowed to attach and spread for 4 hours. Cells were fixed for 30 minutes in 2% paraformaldehyde (in a buffer containing 20 mM PIPES (pH 7.1), 127 mM NaCl, 5 mM KCl, 1.1 mM NaH_2_PO_4_, 0.4 mM KH_2_PO_4_, 2 mM MgCl_2_, 5.5 mM glucose, 1 mM EGTA), then permeabilized in 0.4% Triton X in phosphate-buffered saline (PBS). Cells were blocked for 1 hour in 1% bovine serum albumin in PBS, then immunostained for paxillin with detection by Cy3-conjugated secondary antibody. The coverslips were mounted in Prolong Gold Mounting Media (Invitrogen). Images were acquired using a Nikon Eclipse 80i microscope equipped with a CoolSNAP ES camera (Photometrics) and Plan Apo 60x objective lens.

**FA Area Quantification**

Single cells with a clear polarized morphology were selected for analysis. Background in the nuclear region was eliminated to facilitate maximal FA detection, then initial FA masks were generated using the Moments filter in the ImageJ Segmentation, Multithresholder plugin. To eliminate noise and cellular background, particles in the mask were filtered to remove particles less than 15 pixels (0.344 µm^2^) and greater than 10000 pixels. The remaining particles in the mask were then compared to the original image. To produce the final mask, the particles were subdivided if multiple adhesions were clearly represented within a single particle, or they were deleted if they did not correlate with a clearly visible FA. Finally the area of all particles in the mask was measured using ImageJ particle analysis.
